# Viral fusion efficacy of specific H3N2 influenza virus reassortant combinations at single-particle level

**DOI:** 10.1038/srep35537

**Published:** 2016-10-18

**Authors:** Hung-Lun Hsu, Jean K. Millet, Deirdre A. Costello, Gary R. Whittaker, Susan Daniel

**Affiliations:** 1 School of Chemical and Biomolecular Engineering, Cornell University, Ithaca, NY, USA; 2 Department of Microbiology and Immunology, Cornell University, Ithaca, NY, USA.

## Abstract

Virus pseudotyping is a useful and safe technique for studying entry of emerging strains of influenza virus. However, few studies have compared different reassortant combinations in pseudoparticle systems, or compared entry kinetics of native viruses and their pseudotyped analogs. Here, vesicular stomatitis virus (VSV)-based pseudovirions displaying distinct influenza virus envelope proteins were tested for fusion activity. We produced VSV pseudotypes containing the prototypical X-31 (H3) HA, either alone or with strain-matched or mismatched N2 NAs. We performed single-particle fusion assays using total internal reflection fluorescence microscopy to compare hemifusion kinetics among these pairings. Results illustrate that matching pseudoparticles behaved very similarly to native virus. Pseudoparticles harboring mismatched HA-NA pairings fuse at significantly slower rates than native virus, and NA-lacking pseudoparticles exhibiting the slowest fusion rates. Relative viral membrane HA density of matching pseudoparticles was higher than in mismatching or NA-lacking pseudoparticles. An equivalent trend of HA expression level on cell membranes of HA/NA co-transfected cells was observed and intracellular trafficking of HA was affected by NA co-expression. Overall, we show that specific influenza HA-NA combinations can profoundly affect the critical role played by HA during entry, which may factor into viral fitness and the emergence of new pandemic influenza viruses.

Influenza A viruses are enveloped, single-stranded, segmented, negative-sense RNA viruses that infect a wide variety of bird and mammalian species. The extensive and dynamic genetic diversity of influenza viruses is driven by the frequent generation of mutations due to the error-prone viral RNA polymerase and the process of reassortment of gene segments from distinct viruses. Seasonal human influenza epidemics result in acute respiratory infections that circulate worldwide and cause up to 500,000 deaths each year^1^. Moreover, although they are rare, human influenza pandemics can occur when novel subtypes of influenza viruses emerge through genetic mutations and reassortment. In these situations, high transmissibility and pathogenicity leads to elevated human infections and deaths, as observed during the 1918 pandemic that led to an estimated 50 million deaths worldwide^2^,^3^. The 1957 and 1968 pandemics, caused by the reassortment of avian and human viruses, and more recently, the 2009 pandemic, caused by a triple reassortant between avian, swine, and human viruses highlight the importance of the gene reassortment process in generating influenza virus strains that exhibit markedly altered tropism, pathogenicity, and interspecies transmission characteristics^4^,^5^.

Influenza A viruses require hemagglutinin (HA) and neuraminidase (NA) envelope glycoproteins for cell entry and egress, respectively. HA is a trimeric membrane-embedded glycoprotein that is a critical determinant of host tropism, and mediates both binding to host cell surface sialic acid receptors and fusion of viral and host cell membranes. Because of its crucial role in governing cell entry, tissue and host tropism, HA is also a key factor in regulating viral pathogenicity. NA protein is a tetramer on the surface of virions and is responsible for catalyzing cleavage of terminal sialic acids from HA^6^,^7^. NA’s functional role is critical for the release of progeny viral particles from infected cells at late stages of the infection cycle^8^, and prevents virus from binding to the mucus overlying the human airway epithelium^9^. Influenza A viruses are divided into subtypes based on antigenic and amino acid sequence differences of the HA and NA surface glycoproteins, each composed of 18 and 11 known subtypes, respectively. While different HA and NA combinations are found in circulating influenza viruses, aquatic birds can be infected by most subtypes, while humans are known to be infected by three main subtypes: H1N1, H2N2, and H3N2^10^. The human H2N2 subtype is not currently circulating, but was responsible for the 1957 pandemic^11^. The avian H5N1 and H9N2 subtypes have been recognized for their pandemic potential in the human population^12^. The transition of receptor usage from avian-like α2,3-linked to human-like α2,6-linked sialic acids represents a critical step for avian viruses to acquire efficient replication and transmission capabilities in humans. Furthermore, understanding the effects of switching combinations of gene segments that occur during reassortment, in particular those encoding HA and NA, is critical for uncovering the basis of emergence of influenza viruses with increased pathogenicity and for pandemic preparedness.

During influenza virus entry, which occurs through the endocytic pathway, conformational changes of the HA are critical for virus fusion to occur. HA is a type I transmembrane protein and represents a prototypical class I viral fusion protein that has been widely studied both structurally and functionally. HA is initially synthesized as an uncleaved precursor, HA_0_, which is proteolytically processed by host cell proteases into two subunits, HA_1_ and HA_2_, linked by a single disulphide bond. The HA_1_ subunit contains a globular domain with residues responsible for binding to sialic acids, while the core of the fusion machinery, the hydrophobic fusion peptide, is found within the membrane anchored HA_2_ subunit^13^,^14^. The proteolytic cleavage event is critical for activating the HA, as it allows for exposure of the fusion peptide. In the late endosome, the pH drops from pH 6.5-6 to pH 5-4.5 causing the HA_2_ fusion domain to undergo major conformational changes exposing the fusion peptide and enabling it to be inserted into the target endosomal membrane. HA_2_ then refolds, and pulls viral and endosomal membranes together, allowing for hemifusion to occur and ultimately leading to the opening of the fusion pore and release of the viral genome^15^,^16^. In previous studies, we and others have successfully applied a single-particle tracking (SPT) methodology combining total internal reflection microscopy (TIRFM), microfluidics, and supported lipid bilayers to study influenza HA-mediated fusion kinetics using native viruses or HA-pseudotyped viral particles^17^,^18^,^19^,^20^.

While influenza virus fusion has been well studied, including using the SPT approach, the influence of NA on HA fusion function is less clear. In particular, the comparison of different HA-NA pairings has not been well characterized in the context of HA-mediated fusion. Here, using SPT and a vesicular stomatitis virus (VSV)-based pseudotyping system, we investigated how co-incorporation of NA with HA in viral particles affects HA-mediated fusion kinetics. The VSV-based pseudotyping approach allowed us to focus specifically on the effects of HA-NA pairings. We examined the effects of incorporation of the H3 HA and N2 NA of the prototypical X-31 strain and compared them with authentic X-31 influenza virus fusion kinetics. In addition, to model reassortment of N2 NA, we studied how co-incorporation of heterologous human (H2N2 Japan) or avian (H9N2 MS96) N2 proteins impacts H3 HA-mediated fusion kinetics. The N2 NA gene of the H3N2 (X-31) subtype is genetically more closely related to the one from the H2N2 (Japan) subtype (94.5% amino acid identity) than the one from the H9N2 (MS96) subtype (90.5% identity, with 84 amino acid C-terminal deletion) (Fig. 1). We show that co-incorporation in pseudotyped particles of influenza X-31 H3 and N2 allows faster hemifusion kinetics than particles incorporating only H3. Further, the influenza X-31 H3 and N2 pseudotyped particles exhibited fusion kinetics that closely matched the ones observed for authentic X-31 virus. We also demonstrated that co-incorporation of X-31 H3 with heterologous N2 from the human H2N2 (Japan) or from the avian H9N2 (MS96) are associated with markedly decreased fusion kinetics, results that were consistent with viral phylogenetic relationships, suggesting that even within a given subtype there is some degree of constraint imposed by HA-NA pairings with respect to HA-mediated function. Finally, we show that the differences in hemifusion kinetics we observed could be attributed to changes in relative incorporation of HA and NA in viral pseudotyped particles.

## Results

### Influenza pseudotyped particle production and infectivity assays

To assess the effect of HA-NA pairings on infectivity of influenza pseudotyped viruses, six types of pseudotyped particles were produced: HA only pseudoparticles (H3_X-31_), native matching pseudoparticles (H3_X-31_/N2_X-31_), human:human mismatching pseudoparticles (H3_X-31_/N2_Japan_), human:avian mismatching pseudoparticles (H3_X-31_/N2_MS96_), and two control pseudovirions, VSV-∆env and VSV-G.

**Figure 2:**
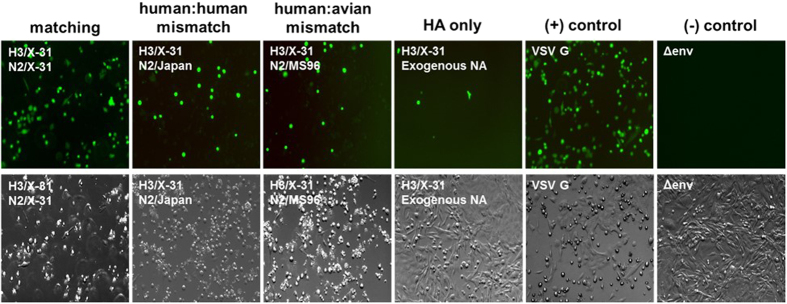
Pseudotyped particle infectivity assay. Top row: MDCK cell infectivity assay of influenza pseudotyped particles H3_X-31_/N2_X-31_, H3_X-31_/N2_Japan_, H3_X-31_/N2_MS96_, H3_X-31,_ VSV and Δenv. Green fluorescence indicates infection of the cell. Bottom row: bright field images of the images above. Images acquired using a 20 × objective.

The pseudovirions were then used to infect Madin-Darby canine kidney epithelial (MDCK) cells, with infection confirmed by the green fluorescence signals produced by the infected cells. In all cases, MDCK cells were evenly seeded at 2 × 10^5^ cells/cm^2^, so more GFP-positive cells indicate that pseudovirions had higher infectivity (Fig. 2). VSV-Δenv refers to particles without envelope glycoproteins (negative control case) and VSV-G are particles harboring the VSV G surface fusion protein (positive control case). The results of the infectivity assays show that VSV-H3_X-31_/N2_X-31_, VSV-H3_X-31_/N2_Japan_ VSV-H3_X-31_/N2_MS96_ and VSV-H3_X-31_ particles are infectious, as GFP-positive cells were observed in all these cases. However, among these, infectivity levels varied based on the number of GFP-positive cells observed. To better understand the basis for the differences in infectivity observed, we carried out single-particle fusion assays to determine if there were differences in cell entry behavior among these particles.

### Single-virion fusion experiments

**Figure 3:**
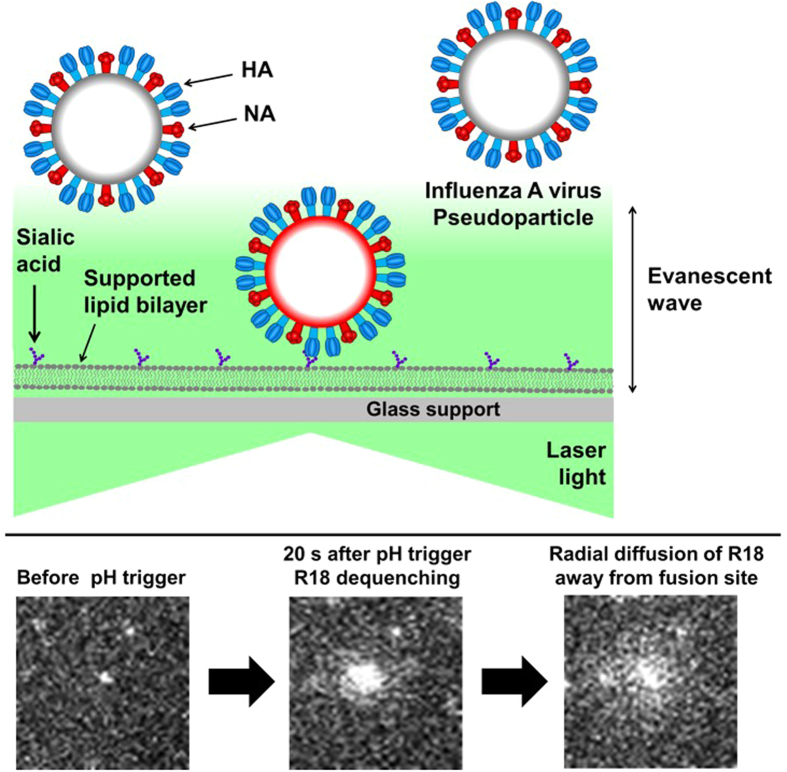
Diagram of single-fusion assay performed with total internal reflection fluorescence microscope (TIRFM). Top illustration: three pseudovirus particles are shown containing HA (blue) and NA (red) in their membrane envelopes. The center virus is bound to sialic acid (purple) in the supported lipid bilayer (gray) localizing the virus in the ~100 nm evanescent wave (green) that results from the total internal reflection of laser light at the glass-buffer interface. The evanescent light excites the fluorophores in the viral membrane, emitting in red. Viruses outside the evanescent field are not excited, and thus no red emission is observed. Note that a membrane-bound pH sensor (Oregon green DHPE) is not shown for clarity. Bottom row: images of a native X-31 virus labeled with R18 fusing with a supported bilayer after triggering with pH 4.5.

To monitor virus entry and fusion at the single particle level, individual virion fusion measurements were performed using total internal reflection fluorescence (TIRF) microscopy. Microfluidic channels were coated with supported lipid bilayers (SLB) (Fig. 3). The SLB contained a mixture of zwitterionic lipids, cholesterol, total ganglioside extract (TGE), and Oregon green DHPE^19^,^21^. TGE contains glycolipids that possess sialic acid groups necessary for influenza binding. Oregon green DHPE is a pH-sensitive fluorophore embedded in the SLB that drastically decreases in emission intensity when exposed to an acidic solution, marking the time when HA-activating acidification occurs in the microfluidic channel. The fluidity of SLBs was confirmed by using fluorescence recovery after photobleaching (FRAP) performed prior to the fusion assay. Membrane fluidity of SLBs can affect the hemifusion kinetics and is thus a crucial parameter to control when comparing the rate of fusion between assays^17^,^21^. The average diffusion coefficient for three samples was determined to be 0.847 ± 0.03 μm^2^/s which corresponds to the value measured in a previous study^21^.

HA-mediated membrane fusion is a multistep reaction that begins with hemifusion (the merging of the outermost leaflets of the host and viral membranes). Hemifusion commences when hemagglutinin is exposed to an acidic solution, which triggers major conformational changes of the viral envelope protein. Pseudovirions were treated with TPCK-trypsin for 15 min at 37 °C in order to cleave the precursor HA_0_ into HA_1_ and HA_2_, which is an essential activating step for successful membrane fusion to occur^13^. To track the fusion reaction and its intermediate steps, viral membranes were labeled with quenched amount of octadecyl rhodamine B chloride (R18). Upon membrane hemifusion, the membrane dye that originated in the viral envelope spreads into the SLB and dequenches as the fluorophores diffuse radially away from the hemifusion site, an event that is easily detected by a CCD camera (Fig. 3, bottom). Fusion experiments were carried out within a channel for 3–4 min at a data collection rate of 10 frames/s.

The hemifusion lag time is defined as the interval of time between the pH drop and hemifusion for each individual virion. Within the field of view, hemifusion is marked by individual dequenching events. The lag time distributions are fit to the cumulative gamma distribution:


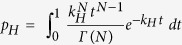


where *k*_*H*_ is the hemifusion or pore formation rate constant, *t* is lag time, and *N* is an additional fit parameter reporting the number of rate-limiting steps. In the context of fusion kinetics, *N* is often correlated to the number of HA trimers that must act concertedly to initiate fusion^19^, but is mathematically defined as the number of steps in the pathway. The gamma distribution is appropriate here, as fusion events occur stochastically and independently of each other. Each fusion event is a multistep process, with each step in the process being described by a Poisson process. The gamma distribution is a convolution of multistep Poisson processes.

**Figure 4:**
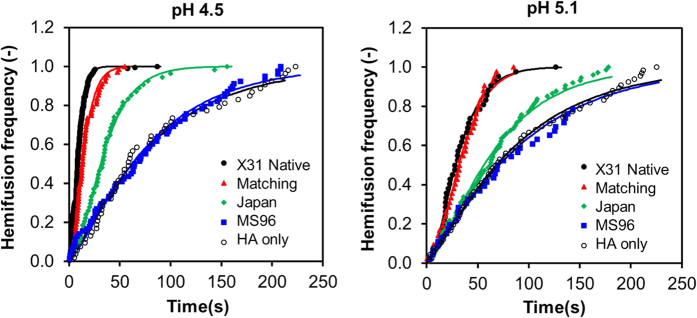
Hemifusion frequency analysis. Cumulative distribution function plot of different batches of native influenza X-31, matching pseudoparticles (H3_X-31_/N2_X-31_), mismatching pseudoparticles (H3_X-31_/N2_Japan_), mismatching pseudoparticles (H3_X-31_/N2_MS96_), and HA only pseudopaticles (H3_X-31_) at pH 4.5 and 5.1.

We characterized the hemifusion behavior of native influenza X-31, native-matching pseudoparticles (H3_X-31_/N2_X-31_), human:human mismatching pseudoparticles (H3_X-31_/N2_MS96_), human:avian mismatching pseudoparticles (H3_X-31_/N2_Japan_), and HA only pseudoparticles (H3_X-31_) over a range of initiation pHs. Figure 4 illustrates hemifusion rates by showing the cumulative gamma distribution data at the upper and lower limits of HA conformational change activation, pH 4.5 and pH 5.1, respectively. At pH 4.5 and 5.1, the rate of hemifusion of native influenza X-31 and matching pseudoparticles (H3_X-31_/N2_X-31_) are similar and are distinctively faster than that of other pseudoviruses, including the HA only pseudoparticles (H3_X-31_).

**Figure 5:**
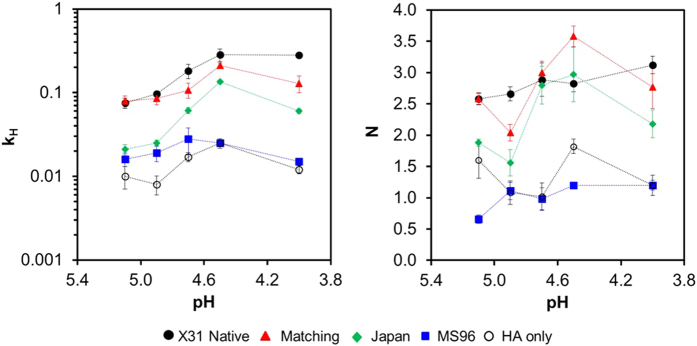
HA hemifusion kinetics. Hemifusion rate constant, *k*_*H*_ and number of HA trimers, *N*, as a function of pH for native influenza X-31, matching pseudoparticles (H3_X-31_/N2_X-31_), mismatching pseudoparticles (H3_X-31_/N2_Japan_), mismatching pseudoparticles (H3_X-31_/N2_MS96_) and HA only pseudoparticles (H3_X-31_). The error bars of the data represent the standard deviation of at least three separate experiments. Dashed lines serve to guide the eye only.

The hemifusion kinetics over a range of initiation pHs between pH 4.0 to pH 5.1 were determined (Fig. 5). The hemifusion rate constants of samples at the tested pH in ascending order are HA only pseudopaticles (H3_X-31_), mismatching pseudoparticles (H3_X-31_/N2_MS96_), mismatching pseudoparticles (H3_X-31_/N2_Japan_), matching pseudoparticles (H3_X-31_/N2_X-31_) and native influenza X-31. The hemifusion rate constants of matching pseudoparticles (H3_X-31_/N2_X-31_) and native influenza X-31 are similar at all initiation pHs. The hemifusion kinetics of native influenza viruses is strongly dependent on pH as well as matching pseudoparticles (H3_X-31_/N2_X-31_). The rate of hemifusion increases almost linearly from pH 5.3 to 4.7 and reaches a plateau at the “fastest” fusion pH. It is not evident from the data shown in Fig. 5 that mismatching pseudotyped particles (H3_X-31_/N2_MS96_) exhibits the same dependence on pH. While the maximum rate of fusion of mismatching pseudovirions (H3_X-31_/N2_MS96_) also occurs at pH 4.7, the error associated with the parameter falls within the rate constant values associated with adjacent pHs, thus it can be concluded that the hemifusion kinetics of mismatching pseudotyped particle (H3_X-31_/N2_MS96_) is triggered by acidification but rate independent of triggering pH. pH independent influenza fusion rate is not unprecedented, as we also observed a similar flat trend for a clinical isolate, A/Brisbane/2007/H3N2^17^.

The number of HA trimers (*N*) required for pseudotyped particle fusion was also calculated and compared with that of native influenza X-31 (Fig. 5). At high, physiological pH values, *N* values for influenza virus X-31 and matching pseudovirions (H3_X-31_/N2_X-31_) are found to be approximately 3, while the *N* values of human:human mismatching pseudovirions (H3_X-31_/N2_Japan_) vary between 1.5 to 3. A few possible interpretations of this variation include a change in the number of HA contributing to bending the membrane over this range, but this is hard to rationalize with our understanding of the fusion process. It may reflect that other processes are dominating the kinetics in this regime, but it also seems at odds with what we know about hemifusion, that there would be such oscillations among rate limiting steps within this range. What seems most likely to us is considering that N is an exponential fit parameter; it gives rise to an inherent sensitivity in data fluctuations and thus produces a wider range of output values. Considering that the authentic X-31 does not have such variation in its fits for N, we may attribute these fluctuations as resulting from variations among the particles themselves: the HA incorporation in VSV pseudotypes are lower than the authentic influenza X-31, or that the morphology of the rhabdoviral pseudovirions are bullet-shaped compared to the spherical morphology of authentic X-31. For human:avian mismatching pseudotyped particles (H3_X-31_/N2_MS96_) and HA-only pseudopaticles (H3_X-31_), *N* is consistently closer to unity as a function of pH. These findings suggest that only one HA trimer may be needed for mismatching pseudotyped particle fusion. However, since hemifusion is a multi-step process, an *N* value equal to 1 more likely indicates that another process less dependent on viral particle properties, e.g. the merging of membranes, may occur so slowly that kinetics are dominated by this single step, thus resulting in a gamma distribution where *N *= 1^22^.

### Incorporation of HA and NA in pseudovirions

**Figure 6:**
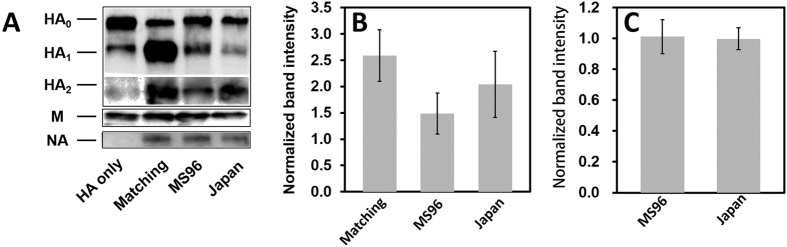
Pseudotyped particle protein density analysis. Quantification of intensities of HA, NA and VSV M protein by Western Blot (**A**). The HA_1_ (**B**) and NA (**C**) band intensities were directly detected by Chemidoc XRS+ imager and normalized to corresponding M band intensity to obtain the relative HA and NA expression levels on different VSV pseudotyped particles. Each error bar represents the mean ± SD of three independent experiments. NA protein was also detected except for HA only particles.

Previous work done by Bosch *et al*., has demonstrated that the presence and activity of NA was key to HA incorporation into lentiviral-based pseudotyped particles and their release from the cell surface^23^. In order to understand better the differences in fusion kinetics observed among matching (H3_X-31_/N2_X-31_) and mismatching pseudovirions, quantitative Western blots were performed to determine the relative protein incorporation of HA in particles. The internal VSV matrix protein (M protein, ~37 kDa) was used to normalize band intensities of HA. The three hemagglutinin bands include one uncleaved HA (HA_0_) at 75 kDa and the cleaved HA (HA_1_ and HA_2_) at 50 kDa and 25 kDa, respectively (Fig. 6). HA_1_ bands were normalized to corresponding VSV M bands to calculate the cleaved proportion of HA protein in different samples. The normalized bands were normalized again to the HA_1_ bands of HA only pseudoparticles (H3_X-31_) so the values could be compared between different HA-NA combinations. HA band intensities of human:avian mismatching pseudoparticles (H3_X-31_/N2_MS96_) and human:human mismatching pseudoparticles (H3_X-31_/N2_Japan_) are ~45% and ~20% lower than X-31 matching pseudoparticles (H3_X-31_/N2_X-31_), respectively. Considering that a successful viral fusion event may require at least three HA trimers, a lower density of HA trimers on mismatching pseudoparticles may explain the slower fusion rate we have measured.

N2 bands (~55 kDa) were detected by Western blot (except in HA only pseudoparticles (H3_X-31_)). Three N2 bands were normalized to corresponding VSV M bands to calculate the NA density on different samples. The normalized bands were normalized again to the matching pseudovirions (H3_X-31_/N2_X-31_), and the quantitative analysis indicated that normalized N2 bands intensity were similar in the three pseudoparticle types containing it.

### HA and NA expression in transfected cells

**Figure 7:**
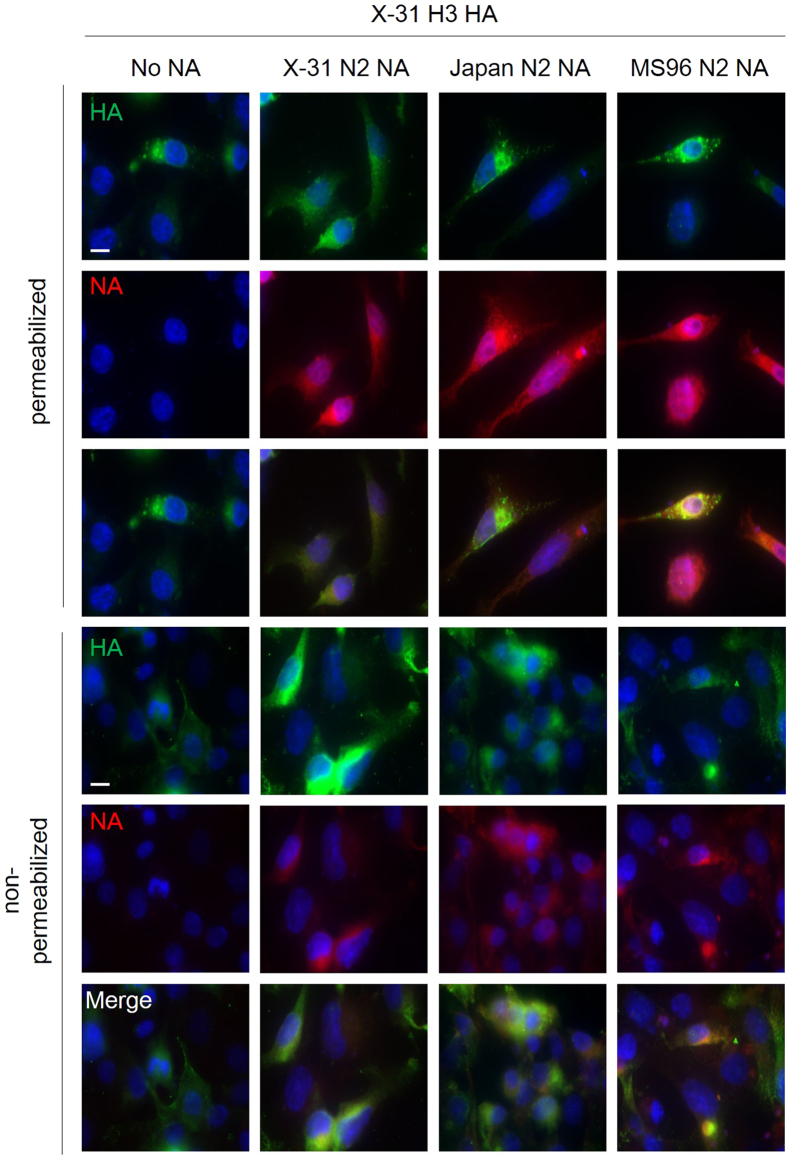
Expression and subcellular localization of HA and NA in HA-NA co-transfected BHK-21 cells by immunofluorescence microscopy assay. Cell nuclei were labeled with DAPI (blue), and HAs/NAs were labeled with anti-HA (clone HA-7) and anti-N2 (H6N2) primary antibodies and with corresponding fluorophore-conjugated antibodies (false-colored with HA in green and NA in red). Top three rows: permeabilized BHK-21 cells imaged using 100 × objective. Bottom three rows: non-permeabilized BHK-21 cells using 100 × objective and with identical exposure time. For each panel, cells were co-transfected with pCAGGS-H3(X-31) and with either (from left to right): pCAGGS-empty (No NA), pCAGGS-N2 (X-31), pCAGGS-N2(Japan) (Japan N2 NA), and pCAGGS-N2(MS96) (MS96 N2 NA). Scale bar represents 10 μm.

During production of pseudoparticles, cells were transfected with the same amount of HA- and NA-encoding plasmids DNA (6 μg), but HA incorporation levels in each type of particle varied, while NA stayed relatively constant, as shown in the Western blot analysis^24^. Hence, an immunofluorescence assay was used to monitor HA and NA trafficking in BHK-21 cells. 24 h post transfection, HA, NA, and cell nuclei were fluorescently labeled in permeablized and non-permeablized conditions (Fig. 7). Staining in the permeablized condition gives an assessment of the HA and NA localization in the cell, while the non-permeabilized condition shows cell surface expression of the viral proteins.

In the permeablized condition (top three panels), HA accumulated mostly within perinuclear vesicles in HA-only (H3_X-31_), mismatching (H3_X-31_/N2_MS96_) and mismatching (H3_X-31_/N2_Japan_) conditions. In matching transfected cells (H3_X-31_/N2_X-31_), HA was found to accumulate in perinuclear patches that extended throughout the cytoplasm. The NAs were found being expressed more diffusely in cells than X-31 HA. No NA signal was observed in the HA-only (H3_X-31_) condition, as expected.

In the non-permeablized condition (bottom three panels), the different NAs and the X-31 HA could be observed co-localizing at the cell surface in most cells. Cells co-transfected with matching H3_X-31_/N2_X-31_ displayed the strongest cell-surface expressed HA labeling. H3_X-31_/N2_Japan_ co-transfected cells had slightly less bright cell-surface HA labeling, followed by H3_X-31_/N2_MS96_ co-transfected condition and cells without NA transfection had the lowest cell-surface HA expression.

Overall, the immunofluorescence microscopy results are in alignment with the report by Galloway *et al*. which also compared the expression of H3 in X-31 HA-transfected cells with and without cognate N2, and showed that NA influences trafficking of HA to the cell surface in BHK and Vero cells^25^. Taken together, these results indicate that the matching NA is required for efficient HA trafficking to the plasma membrane, which is the site of virion budding for both native influenza and VSV pseudotyped particles. Danieli *et al*. and Lee *et al*. suggest that an influenza A virus requires at least three HA trimers to be close to each other to form a hemifusion site^22^,^26^, which indicates that the density of HA trimers affects the hemifusion rate of influenza A virus. The immunofluorescence analysis shown here confirms the Western blot HA densitometry data and suggests that NA plays an important role in expression and intracellular trafficking of HA, and that even within the same subtype (N2), different NAs can influence cell-surface expression of HA.

### Effect of NA inhibition on hemifusion kinetics

Influenza NA has an important role during progeny virus egress from host cell by enzymatically cleaving sialic acids and facilitating the release of viral particles^15^,^27^,^28^. Here, our immunofluorescence assay result suggests that heterologous N2 proteins modify H3 trafficking in HA-NA co-transfected cells, and can lead to a decrease in HA incorporation levels in VSV pseudotyped particles. Single-particle fusion analyses reveal that the rate of hemifusion decreased significantly with lower surface HA protein expression level^29^.

**Figure 8:**
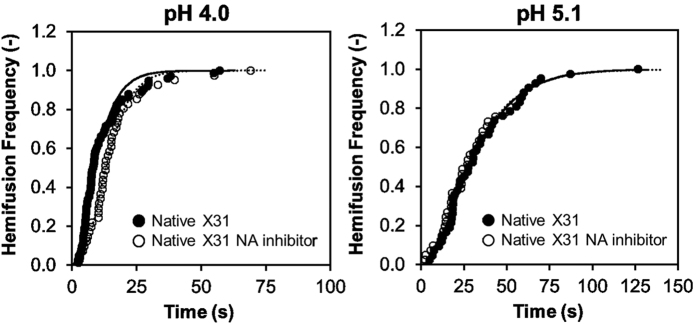
Effect of NA activity inhibition on hemifusion frequency of influenza virus X-31. Cumulative distribution of hemifusion events at pH 4.0 and pH 5.1 for NADNA-treated influenza X-31 and non-treated influenza X-31.

In order to assess whether the enzymatic activity of NA plays a role in these observations, we used N-Acetyl-2,3-dehydro-2-deoxyneuraminic acid (NADNA), a known inhibitor of NA, to block the neuraminidase activity on influenza X-31 (Fig. 8). The data demonstrates the hemifusion kinetics of NADNA-treated influenza virus and non-treated virus are similar at pH 4.0 and pH 5.1, and there were no significant differences on viral binding either. The hemifusion rate constant of NA inhibitor treated virus is 0.24 s^−1^ with a value of 2.5 for N at pH 4, and 0.06 and 1.9 at pH 5.1. That NADNA does not affect hemifusion kinetics of the virus follows the conclusions of Ohuchi *et al*.’s work^29^, where they also found that NA inhibitors do not impact either binding or fusion; however, they suggest that NA function does impact endocytosis, and in this way, impacts “entry”. Since the SPT assay presented here isolates binding and fusion processes from endocytosis, our results corroborate Ohuchi *et al*.’s observations and reinforces our conclusion that differences in expression level (HA/NA) balance among particles is important for the fusion process.

## Discussion

The ever-evolving diversity of influenza virus strains, particularly those of avian origin, represents a pressing concern for global health. While it is well known that mutations and the reassortment of gene segments generates this diversity, understanding the consequences of different combinations of viral genes, particularly those encoding HA and NA still awaits further elucidation. Some studies have shown that a functional balance of HA and NA is required for virus replication^30^ and that insufficient NA enzymatic activity leads to the formation of virus aggregates on egress^31^. Here, we have investigated how incorporation of different NAs affect the fusion function of the HA protein and provide more evidence for a potential role for NA during entry and glycoprotein transport.

Using a VSV-based viral pseudotyping approach we were able to specifically study the interplay between HA and NA. This system allowed us to study the co-incorporation of H3 HA and N2 NA of the prototypical strain X-31 and to switch N2 NAs of different strains. We produced four different types of influenza pseudoparticles: pseudoparticles containing HA only (H3_X-31_), X-31 HA and NA matching pseudoparticles (H3_X-31_/N2_X-31_), and to model N2 NA gene reassortment, human:human mismatching pseudoparticles (H3_X-31_/N2_Japan_) and human:avian mismatching pseudoparticles (H3_X-31_/N2_MS96_). Particles that incorporated both X-31 HA and NA displayed distinctly faster fusion kinetics than those harboring HA only. Further, we show that the X-31 matching pseudoparticles (H3_X-31_/N2_X-31_) exhibit similar hemifusion kinetics than native influenza X-31. Other combinations of particles display different kinetics from the native particles.

The significant decrease of hemifusion rate between influenza X-31 and mismatching pseudoviruses could be explained by the variations in HA incorporation on the pseudotyped particles when different NAs were co-transfected^23^,^25^, a hypothesis confirmed by quantitatively analyzing HA_1_ bands in Western blot assays performed on concentrated pseudotyped particles. However, the degree of NA incorporation by Western blot showed that the densities were similar for the three different NAs in the pseudoparticles. These results are also supported by the immunofluorescence microscopy assay that shows that HA trafficking varies considerably depending on which NA is co-expressed in transfected BHK-21 cells, leading to differential HA expression on cell plasma membranes, whereas NA expression does not seem as affected. These analyses demonstrate that NA does not act directly on HA function but rather has an indirect effect by modulating the trafficking and incorporation of HA into pseudotyped viral particles. Our work shows that switching NAs of the same subtype (N2) can have profound effect on fusion kinetics mediated by the HA protein. This study suggests that HA-NA combinations form pairs of varying compatibility that can ultimately impact HA fusogenicity. In summary, we show that influenza X-31 H3 HA requires its matching neuraminidase to maximize the HA packaging density on VSV pseudoparticles, and the matching pseudoparticles exhibit similar hemifusion kinetics as influenza X-31 by single virion fusion assay.

Beyond this study, there are some directions worth pursuing next. First, defining the pore formation of VSV pseudoparticles is an important step to understanding the impact of HA-NA balance on genome transfer. From previous studies we know that the pore formation kinetics of influenza H3N2 is described by a simple single exponential decay (one step transition from hemifusion intermediate to open pore)^17^,^19^ which begs the question if pseudovirus particles behave in the same way, or if the HA-NA balance may impact this process as well. The SPT fusion platform has been useful for screening HA neutralizing antibodies^32^. We note a recent development of a high-throughput, microdroplet-based single particle hemifusion assay^33^,^34^ that is a potentially powerful tool to screen antibodies could be also useful in expanding the studies presented here to characterize many phenotypes quickly, based on hemifusion function and characteristics.

## Materials and Methods

### Cells, plasmids, and viruses

Baby hamster kidney-21 (BHK-21) cells and Madin-Darby canine kidney (MDCK) cells were obtained from the American Type Culture Collection (ATCC, Manassas, VA) and grown in Dulbecco’s modified Eagle medium (DMEM, CellGro), supplemented with 10% fetal bovine serum (Gibco), 1% penicillin and 10 μg/mL streptomycin (CellGro), 1% HEPES buffer (CellGro). The cells were cultured in a 37 °C, 5% CO_2_ incubator.

The plasmids pCAGGS-H3/X-31, pCAGGS-N2/X-31, pCAGGS-N2/MS96, pCAGGS-N2/Japan, pCAGGS-VSVG and pCAGGS-empty were used to transfect BHK-21 cells. pCAGGS-H3/X-31 and pCSAGGS-N2/X-31 encode the hemagglutinin and neuraminidase of influenza virus X-31, respectively. pCAGGS-N2/MS96 encodes the neuraminidase of influenza virus MS96 (H9N2), pCAGGS-N2/Japan encodes the neuraminidase of influenza virus Japan (H2N2), and pCAGGS-VSVG encodes the glycoprotein of vesicular stomatitis virus. The pCAGGS vector serves as the empty vector control. Influenza X-31 A/Aichi/68 H3N2 (Charles River, Wilmington, MA) (live virus) was used as a reference case in the study.

The VSV *G-VSVΔG is a recombinant virus that harbors a genome in which the glycoprotein gene is replaced by the green fluorescence protein (GFP) reporter gene, and is a generous gift from Michael Whitt from University of Tennessee.

### Preparation of influenza pseudoparticles

VSV-based pseudotyped particles were produced as described previously^35^,^36^. 9 × 10^5^ cells BHK-21 were seeded in 10 cm petri dishes (Corning), and incubated for 24 h. BHK-21 cells were transfected by a mixture containing 36 μL of lipofectamine 2000 transfection reagent (Thermo Fisher) and 12 μg of plasmid DNA (6 μg of HA- and NA-encoding plasmids DNA) for each plate, and incubated for 24 h. Next, transfected BHK-21 cells were inoculated with VSV *G-VSVΔG in RPMI medium (10.4 g RPMI powder, 26.7 mL BSA 7.5% liquid, 25 mL 1 M HEPES, 1 L H_2_O), and incubated for 2 h at 37 °C with rocking. Unbound viruses were washed out with DPBS (Dulbecco’s phosphate-buffered saline), and incubated for 24 h at 37 °C with DMEM growth medium. For HA only case, 0.25 units of exogenous neuraminidase from *C. welchii* (Sigma-aldrich) was added to the plate to facilitate viral particle release. The supernatants, which contain pseudotyped particles, were collected after several gentle taps on the walls of petri dishes to help the release of particles. The supernatants were ultracentrifuged in a Ti45 rotor at 35,000 rpm for 120 min. The supernatants were discarded, and pellets were resuspended in 3 μg/ml trypsin and incubated for 30 min at 37 °C to activate HA. The trypsin-treated viral solutions were then aliquoted for storage at −80 °C.

### Western blotting

Pseudotyped particles were concentrated using an ultracentrifuge at 20,000 rpm in a TiSW28 rotor (Beckman-Coulter) for 2 h at 4 °C. The pellets were resuspended in 30 μL of 3 μg/mL trypsin EDTA (Thermo Fisher Scientific) solution, and placed in a 37 °C water bath for 15 min. The samples were then analyzed by Western blot using goat anti-A/HongKong/1/68 (H3) (NR-3118, BEI resources), goat anti-A/shorebird/Delaware/127/1997(N2) (NR-670, BEI resources) and mouse anti-VSV-M antibodies (Kerafast) followed by incubation with HRP-conjugated rabbit anti-goat IgG (Life Technologies) and HRP-conjugated goat anti-mouse IgG (Life Technologies). All Western blots were visualized and analyzed using a Chemidoc XRS+ system with Image Lab image capture software (BioRad). TheImage Lab software was used to quantify intensities of bands using low sensitivity setting. The software detects the bands by the signal contrast between band and background. We note that all bands have only been adjusted in contrast and brightness in the Image Lab software. The software shows the original signal intensities of the bands.

### Immunofluorescence assay

5 × 10^5^ cells BHK-21 cells were seeded in 8-well glass slides (Millipore). After 24 h, BHK-21 cells were transfected by a mixture containing 0.75 μL of lipofectamine 2000 transfection reagent (Thermo Fisher Scientific) and 1 μg of plasmid DNA (500 ng of HA- and NA-encoding plasmid DNA) for each well, and incubated for 24 h. Transfected cells were then fixed with 4% paraformaldehyde (PFA) (Thermo Fisher Scientific) for 15 min and PFA was then quenched in 50 mM ammonium chloride (NH_4_Cl) for 15 min. For the permeabilized condition, 0.1% Triton X-100 was added to each well for 5 min at 4 °C and washed three times with DPBS. The cells then were blocked with 5% normal goat serum for 45 min, and labeled with monoclonal mouse anti-HA antibody (Sigma-Aldrich), and goat anti-A/shorebird/Delaware/127/1997(N2) (NR-670, BEI resources), followed by labeling Alexa Fluor 568-conjugated goat anti-mouse antibody (Thermo Fisher Scientific) and 488-conjugated chicken anti-goat antibody (Thermo Fisher Scientific). The nuclei were labeled with DAPI (Southern Biotech). Microscopy images were acquired using an inverted microscope (Carl Zeiss) with a 100x objective.

### Preparation of liposomes

Liposomes used in this study contain a 4:4:2:0.5:0.01 molar ratio of 1,2,dioleoyl-sn-glycero-3-phosphocholine (DOPC), 1-oleoyl-2-palmitoyl-sn-glycero-3-phosphocholine (POPC), cholesterol, total ganglioside extract (TGE) and Oregon green DHPE, based on compositions found in previous studies that provide comparative data^19^,^21^. The lipids were purchased from Avanti Polar Lipids (Alabster, AL), and Oregon green DHPE was purchased from Molecular Probes, Eugene, OR. To form liposomes, all components were dissolved and mixed in biotechnology grade chloroform (Sigma-Aldrich) in a glass vial. The bulk solvent was first removed by blowing high purity nitrogen and the solution was placed in a desiccator under vacuum for 3 h to ensure complete evaporation of the solvent. GPMV buffer (50 mM Hepes, 150 mM NaCl, 2 mM CaCl_2_, pH 7.4) was then added to the vial to re-suspend the dried lipid film to create a 5 mg/mL solution. Liposomes were then extruded ten times through a 100 nm pore size polycarbonate filter (Whatman Nucleopore), and five times through a 50 nm pore size filter.

### Fluorescent labeling of viruses

Both influenza viruses and the influenza pseudoparticles were labeled with the lipophilic fluorophores: octadecylrhodamine B chloride (R18), a red-emitting fluorophore, at a sufficient concentration and sonicated 30 min to quench fluorescence.

### NA inhibition assay

The neuraminidase inhibitor N-Acetyl-2,3-dehydro-2-deoxyneuraminic acid (NADNA) (Sigma-Aldrich) was dissolved in DI water to 2 mg/mL. 1 μL of NADNA was mixed with 6 μL of influenza X-31 virus and incubated at 37 °C for 1 h. The NADNA-treated virus was labeled as described in the labeling procedure and fusion carried out at selected triggering pH’s.

### Preparation of glass surfaces for supported bilayers

To produce biomimetic planar supported lipid bilayer, various methods have been developed^37^,^38^,^39^. Here, self-assembling of lipid vesicles was performed to form a host cell-mimetic membrane bilayer, and the procedure is described as follows. Glass microscope coverslips (25 mm × 25 mm; No. 1.5) from VWR were cleaned in piranha solution (45 mL 50% hydrogen peroxide and 105 mL 70% sulfuric acid) for 10 min then rinsed 30 min with deionized water with a minimum resistance of 18.2 MΩ cm (Siemens Purelab Ultra water purification system). Glass slides were flushed with deionized water again and dried by a stream of ultra-pure nitrogen gas prior to plasma cleaning.

### Fabrication of microfluidic devices

The generation of microchannel silicon mold developed using soft lithography was published previously^18^,^21^,^39^. The pattern contains six trenches 70 μm deep, 135 μm wide and 1.5 cm long with 100 μm spacing between each channels. Microfluidic devices were formed using polydimethylsiloxane (PDMS) in a molding process. The silicon mold was coated with chlorotrimethylsilane (Sigma-Aldrich) via vapor deposition to facilitate the release of cured PDMS. A 10:1 (elastomer:crosslinker) mixture of Sylgard 184 (Dow Corning) was mixed and degassed to remove bubbles before pouring on the silicon mold. The PDMS then was crosslinked in the oven for 3 h at 80 °C. Both the piranha cleaned glass cover slip and the microfluidic device were assembled by oxygen plasma bonding. They were first treated with oxygen plasma using a Harrick Plasma Cleaner (Model # PDC-32G, Ithaca, NY) at a pressure of 750 millitorr on the “high” setting for 15 s. The two pieces were then pressed together gently to form a tight bond and annealing was performed at 80 °C for 15 min.

### Forming supported bilayers in microfluidic channels

A 10% dilute solution of liposomes was drawn into the microchannel at a flow rate of 100 μL/min for 90 s and incubated on the glass substrates for 2 h. The microchannel was then rinsed with the GPMV buffer at 100 μL/min for 2 min. To heal defects in membranes, a 5% dilute solution of liposomes was drawn into the microchannel at 10 μL/min for 5 min. Before loading virus-containing solutions, channels were rinsed again with GPMV buffer at 100 μL/min for 2 min.

### TIRF microscope configuration

Single-particle fusion assays were operated using total internal reflection fluorescence microscopy (TIRFM) with an inverted Zeiss Axio Observer.Z1 with an α Plan-Apochromat 100× oil immersion objective with a numerical aperture (NA) of 1.46. Index-matching immersion oil (Carl Zeiss, Inc.) was added to the glass coverslip of the microfluidic device and the objective. Two lasers with 561 nm and 488 nm excitation wavelengths were used to simultaneously excite red and green fluorophores under this setting. The Laser TIRF 3 slider (Carl Zeiss, Inc.) was used to control the angles of incidence in the optical pathway. Exceeding the critical angle (~62°) generates total internal reflection due to the difference in refractive indices of the two different substances (glass and aqueous buffer), and further creates an evanescent wave that penetrates about 100 nm into the aqueous buffer. Because virion binding and fusion occurs within a ~100 nm thick region, the evanescent wave can excite fluorophores near the membrane and eliminate the background noise from viruses floating in the bulk aqueous phase.

### Image processing

Membrane fusion events were analyzed using both ImageJ (NIH) and MATLAB (Mathworks). Each fusion event was manually selected in ImageJ with Time series analyzer V2.0 plugin, and the intensity of each event in 4 × 4 pixels was averaged and saved with the corresponding time as a table. The data was then processed by MATLAB to calculate the fusion lag time and used to fit the lag times into a gamma-distribution equation to retrieve the fusion rate constant^19^.

## Additional Information

**How to cite this article**: Hsu, H.-L. *et al*. Viral fusion efficacy of specific H3N2 influenza virus reassortant combinations at single-particle level. *Sci. Rep.* **6**, 35537; doi: 10.1038/srep35537 (2016).

## Figures and Tables

**Figure 1:**
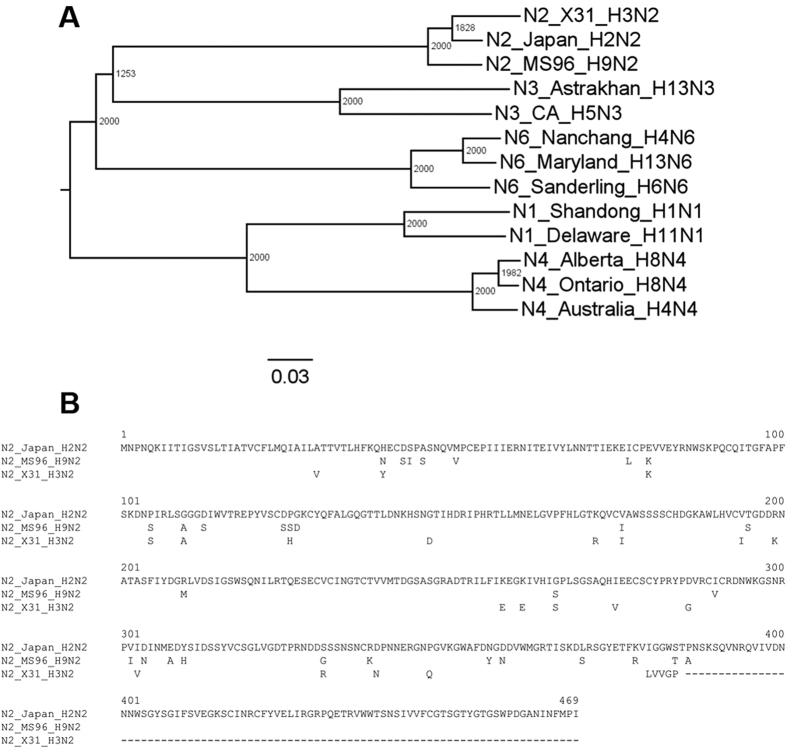
Phylogenetic analysis of influenza A virus NA. (**A**) Phylogeny of influenza NA genes, including N2 genes from A/Aichi/68-X-31(H3N2), A/Japan/305/57 (H2N2) and A/chicken/Korea/MS96/96 (H9N2). The phylogenetic tree was generated using the NA amino acid sequences of isolated influenza A viruses and with alignments performed with ClustalX 2.1 (displayed with FigTree) with the Neighbor-Joining method and bootstrap values calculated from 2000 trees. Scale bar represents estimated number of substitutions per site. Numbers at nodes represent boot strap values. (**B**) Amino acid sequence alignment of influenza A N2. Sequence of A/Aichi/68-X-31(H3N2), A/Japan/305/57 (H2N2) and A/chicken/Korea/MS96/96 (H9N2). Residues identical to N2 (Japan) are left blank. Numbering is based on the N2 (Japan) sequence. The alignment was generated using ClustalW in DNASTAR.
